# Current and upcoming approaches to exploit the reversibility of epigenetic mutations in breast cancer

**DOI:** 10.1186/s13058-014-0412-z

**Published:** 2014-07-29

**Authors:** Fahimeh Falahi, Michel van Kruchten, Nadine Martinet, Geke Hospers, Marianne G Rots

**Affiliations:** 1 Department of Pathology and Medical Biology, University Medical Center Groningen, University of Groningen, Groningen, 9700 RB the Netherlands; 2 Department of Medical Oncology, University Medical Center Groningen, University of Groningen, Groningen, 9700 RB the Netherlands; 3 Institute of Chemistry, UMR CNRS 7272, University of Nice Sophia Antipolis, Nice, Cedex 2 06108 France

## Abstract

DNA methylation and histone modifications are important epigenetic modifications associated with gene (dys)regulation. The epigenetic modifications are balanced by epigenetic enzymes, so-called writers and erasers, such as DNA (de)methylases and histone (de)acetylases. Aberrant epigenetic alterations have been associated with various diseases, including breast cancer. Since aberrant epigenetic modifications are potentially reversible, they might represent targets for breast cancer therapy. Indeed, several drugs have been designed to inhibit epigenetic enzymes (epi-drugs), thereby reversing epigenetic modifications. US Food and Drug Administration approval has been obtained for some epi-drugs for hematological malignancies. However, these drugs have had very modest anti-tumor efficacy in phase I and II clinical trials in breast cancer patients as monotherapy. Therefore, current clinical trials focus on the combination of epi-drugs with other therapies to enhance or restore the sensitivity to such therapies. This approach has yielded some promising results in early phase II trials. The disadvantage of epi-drugs, however, is genome-wide effects, which may cause unwanted upregulation of, for example, pro-metastatic genes. Development of gene-targeted epigenetic modifications (epigenetic editing) in breast cancer can provide a novel approach to prevent such unwanted events. In this context, identification of crucial epigenetic modifications regulating key genes in breast cancer is of critical importance. In this review, we first describe aberrant DNA methylation and histone modifications as two important classes of epigenetic mutations in breast cancer. Then we focus on the preclinical and clinical epigenetic-based therapies currently being explored for breast cancer. Finally, we describe epigenetic editing as a promising new approach for possible applications towards more targeted breast cancer treatment.

**Electronic supplementary material:**

The online version of this article (doi:10.1186/s13058-014-0412-z) contains supplementary material, which is available to authorized users.

## Introduction

Cells in one organism generally contain the same genetic information but present very different gene expression profiles. Epigenetic modifications underlie cell identity by switching genes on or off during mammalian development, without altering the DNA sequence. The heritability of epigenetic modifications plays critical roles in maintaining cell-type-specific gene expression during cell divisions [1]. DNA methylation and histone modification signatures, especially those on promoter regions of genes, are well known to be associated with gene expression.

DNA methylation, the first identified epigenetic modification, is written by a family of DNA methyltransferases (DNMTs). It occurs on carbon 5 of the cytosine mostly in the context of the dinucleotide cytosine phosphate guanine; it is classically known that the DNA methylation status of promoter regions is inversely correlated with gene expression [2]. As such, DNA hypermethylation has been suggested to inhibit expression of retroposons/transposons, and DNA methylation may be involved in establishing as well as maintaining mono-allelic patterns of genes (for example, imprinting and X-chromosome inactivation) [3]. In addition, DNA methylation is thought to be a key player in prevention of chromosomal instability, translocations and gene disruption [1]. DNA methylation was thought to be irreversible until the recent discovery of enzymes that oxidize the methylated cytosine and convert it to hydroxymethyl cytosine, providing intermediates in the process of active DNA demethylation [3],[4].

**Figure 1:**
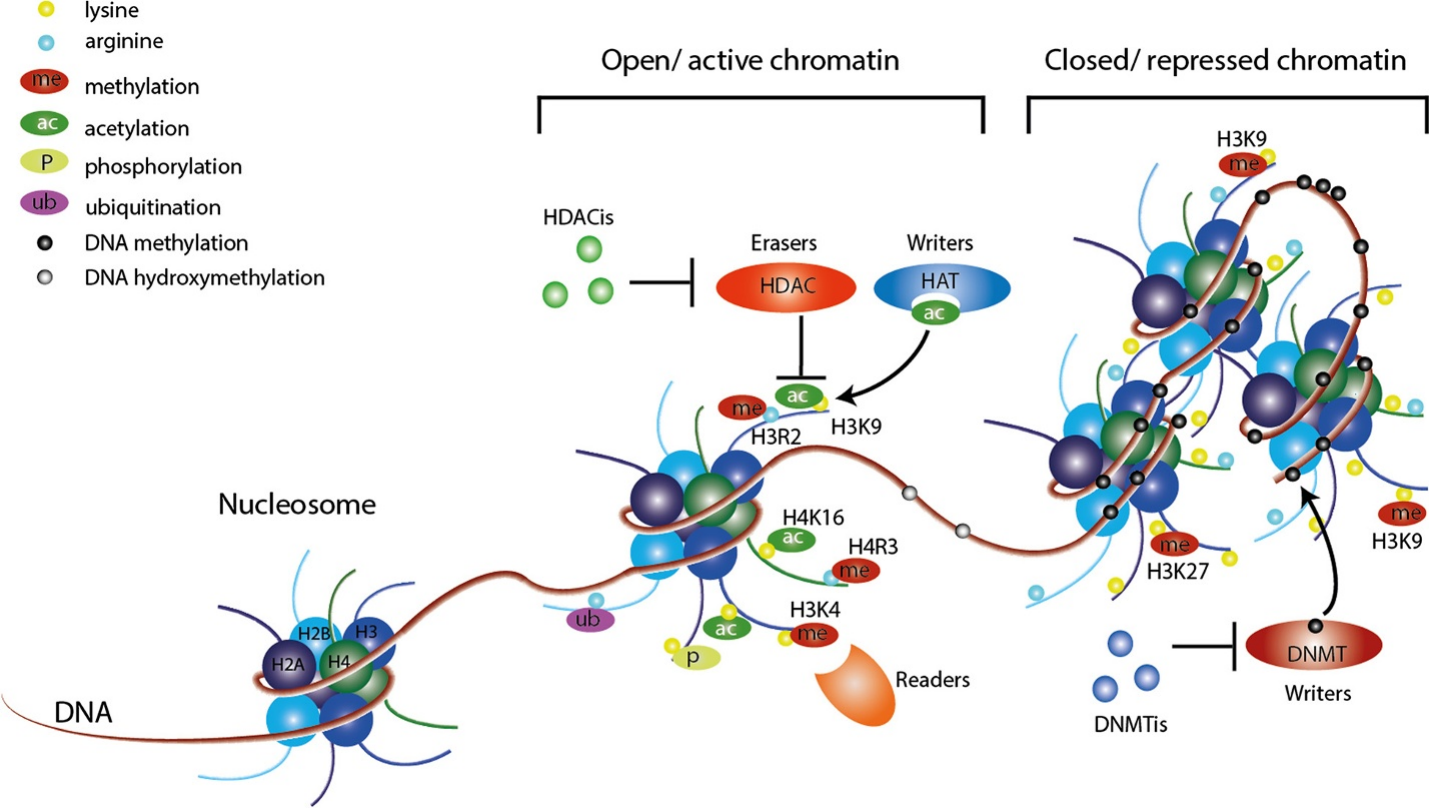
**Epigenetic enzymes and their inhibitors.** The figure shows the interactions between epigenetic enzymes (writers, erasers, readers) and nucleosomes. The nucleosome core consists of a histone octamer (mainly two copies each of H2A, H2B, H3 and H4) that is wrapped by a nuclear DNA strand of 147 bp. DNA methylation and hydroxymethylation are depicted as black and grey circles, respectively. DNA methylation is induced by DNA methyltransferases (DNMTs). To inhibit DNA methylation, DNMT inhibitors (DNMTis) are used to target and suppress DNMTs. Histone tales can be post-transcriptionally modified using enzymes such as histone acetyltransferases (HATs). Histone acetylation can be inhibited by histone deacetylases (HDACs), and HDAC inhibitors (HDACis) can be used as HDAC suppressors.

In addition to DNA methylation, various post-translational histone modifications have been described to be associated with gene expression [1]. In nucleosomes, the histone octamer proteins (generally two copies each of H2A, H2B, H3, and H4) provide the scaffold around which 147 bp of nuclear DNA is wrapped. Histone tails (especially the amino-terminal domains of histones) undergo extensive post-translational histone modifications (for example, acetylation, methylation, ubiquitination, phosphorylation) on some residues, especially lysine and arginine [1] (Figure 1).

Histone modifications as well as DNA methylation are reversible. A very dynamic form of post-translational histone modification is histone acetylation, which mainly occurs on lysine residues and involves histone acetyltransferases (HATs) and histone deacetylases (HDACs) (Figure 1). There are four classes of HDACs with 18 members, HDACs 1 to 11 and Sirtuins 1 to 7. Acetylation of histones reduces their negative charge, thereby, according to early *in vitro* studies, reducing the strength of the histone-DNA interaction and making DNA accessible to transcription factors. Although it is still believed to be involved in regulation of gene transcription, acetylation of histone tails would not be sufficient by itself to regulate gene transcription *in vivo* and in the chromatin context. The effect of histone acetylation on gene regulation is dependent on various factors, including, but not limited to, the position of acetylation [5].

Various epigenetic enzymes are continuously acting to maintain the balance of epigenetic modifications by inducing (‘writers’) or removing (‘erasers’) epigenetic modifications. Other epigenetic players bind to epigenetic modifications (‘readers’) and recruit further re-enforcing complexes (Figure 1). Malfunctioning of these enzymes results in aberrant epigenetic modifications (epigenetic mutations). Since epigenetic enzymes interact with, recruit or suppress each other, while also epigenetic modifications recruit epigenetic enzymes [6], malfunctioning of any epigenetic enzyme can be sufficient to severely affect the epigenome and disrupt the normal state of the cell. The function of epigenetic enzymes is thus vital in maintaining the normal state of cells.

In cancer, numerous epigenetic enzymes are frequently mutated and/or dysregulated, resulting in altered epigenetic modifications [1],[2],[7]–[10]. The dysregulated epigenetic enzymes in cancer are potential targets of several classes of inhibitors, including DNMT inhibitors (DNMTis), HDAC inhibitors (HDACis), and the recently developed inhibitors of histone methyltransferases and HATs. Inhibitors of epigenetic enzymes used in (pre)-clinical treatments are so-called epi-drugs.

## Epigenetics and breast cancer

Extensive studies on epigenome changes in breast cancer have been undertaken to understand the role of epigenetics in breast cancer and to develop novel epigenetic therapies. Such studies have demonstrated the association of aberrant DNA hypomethylation not only with cancer in general, but also with breast cancer [11]. In addition to global blocks of DNA hypomethylation, which underlies chromosomal instability and disturbed gene expression patterns, hypermethylation of promoter regions of, for example, tumor suppressor genes is found in breast cancer [12]. Decreased levels of DNA hydroxymethylation are also observed in breast tumors versus normal breast tissue [13].

Besides the hypermethylated tumor suppressor genes, genes involved in DNA repair, apoptosis, metabolism, cell cycle regulation, cell adherence, metastasis, cellular homeostasis, and cell growth and genes encoding several epigenetic enzymes are frequently hypermethylated in breast cancer [2],[12]. Aberrant DNA hypermethylation of some key genes in breast cancer might be useful as prognostic or diagnostic markers. For instance, aberrant hypermethylation of genes encoding estrogen receptor (ER)-α and progesterone receptor (PR) is correlated with silencing of these genes and with development of ER- and PR-negative breast cancer. Indeed, some hypermethylated genes, such as *RASSF1A*, are considered as potential diagnostic markers of breast cancer [2]. Also, aberrant DNA hypermethylation of the gene *PITX2* (*paired like homeodomain transcription factor-2*) in breast cancer was recently considered as a marker linked to tamoxifen resistance [2]. Thus, the DNA methylation status of such genes might show value as a predictive marker for therapy response.

Another common occurrence in cancer is the global reduction of monoacetylated lysine 16 of histone H4 (H4K16) [13]. The loss or low levels of H4K16 acetylation was suggested as an early event in breast cancer [7],[14] and is associated with altered levels of HDACs [15]. Moreover, mutated HATs have been reported in breast cancer [1]. Altered histone methylation patterns [16] as well as mutated histone methyltransferases are also observed in breast cancer [1].

Altogether, maintenance of the balance of epigenetic modifications by epigenetic enzymes is essential for the regulation of gene expression and the maintenance of the normal status of cells. Clearly, malfunctioning of epigenetic enzymes and the subsequent aberrant epigenetic modifications are involved in development and progression of different cancer types, including breast cancer. Treatments to reverse the aberrant epigenetic modifications are currently under intensive preclinical and clinical investigations and are discussed below.

## Preclinical studies on epigenetic therapy for breast cancer

The reversible nature of epigenetic modifications makes epigenetic mutations attractive targets for epigenetic therapy of cancer. Currently, intensive research is focused on inhibiting epigenetic enzymes such as DNMTs and HDACs. Although aberrant histone methylation modifications occur in breast cancer, to the best of our knowledge there is no report describing the effects of any histone methyltransferase inhibitors on breast cancer. DNMTis and HDACis have been tested as therapeutic interventions against several tumor types, including breast cancer. Here, we discuss the different DNMTis and HDACis and their efficacy in preclinical breast cancer studies.

### DNA methyltransferase inhibitors

DNMTis are used to prevent DNA re-methylation after cell division and can be classified as nucleoside analogues and non-nucleoside analogues. Azacitidine (5azaC, Vidaza®, Celgene Corp., Summit, NJ, USA) and decitabine (5azadC, Dacogen®, SuperGen, Inc., Dublin, CA, USA) are two well-known examples of nucleoside analogues [17]. Both are incorporated into the DNA during replication and, by forming covalent bonds with DNMTs, they trap them and block their functions [17].

Azacitidine is considered a global DNMTi and can be incorporated into both DNA and RNA. For example, upon treatment of breast cancer cells with azacitidine, DNA re-methylation was inhibited for 23 out of 26 tested hypermethylated genes in breast cancer. Further analysis of five selected genes demonstrated their re-expression [18].

Animal studies further validated the potential therapeutic implications of such observations. Assessment of several therapeutic doses of azacitidine showed association of azacitidine with tumor size reduction of xenografts derived from breast cancer cells [19]. In this study, treatment of the immunodeficient mice with 0.5 mg/kg azacitidine for 5 days a week was correlated with growth inhibition of patient-derived tumors that were engrafted orthotopically into these mice [19].

Decitabine treatment also prevents DNA re-methylation and re-activates silenced genes [19]. For example, it was able to induce tumor necrosis factor related apoptosis-inducing ligand (TRAIL) in triple-negative breast cancer cells [20], which can explain how this DNMTi makes breast cancer cells sensitive to chemotherapeutic agents [21]. Decitabine treatment of animals with orthotopically implanted breast cancer cells resulted in reduced tumor volume [22]. Similarly, breast cancer cells pre-treated with decitabine showed diminished tumor growth upon xenografting [19].

Importantly, demethylation and re-expression of genes involved in endocrine therapy response, such as *ESR1* (encoding ER-α), can be exploited to overcome endocrine therapy resistance in ER-negative breast cancer [2]. Such strategies open new possibilities for otherwise difficult-to-treat breast cancers.

Non-nucleoside DNMTis include several classes of natural compounds, such as the polyphenols [17]. Epigallocatechin-3-gallate, a major catechin found in green tea extract, was found to induce apoptosis in breast cancer via inhibiting expression of genes such as *VEGF* (*vascular endothelial growth factor*) [23] and it was shown to induce re-expression of *ESR1* in breast cancer cells [24].

So regardless of the type of agent, inhibition of DNMTs results in re-expression of tumor suppressor genes associated with inhibition of growth of cancer cells.

### Histone deacetylase inhibitors

HDACis chelate the zinc co-enzyme factor, thereby blocking HDACs catalytic activity. HDACis are divided into four groups: short chain fatty acids (for example, sodium butyrate, valproic acid), hydroxamic acids (for example, trichostatin A, vorinostat, panobinostat), cyclic tetrapeptides (for example, depsipetide, romidepsin (isostax)), and benzamides (for example, entinostat, tacedinaline) [20].

HDACis as monotreatment *in vitro* and *in vivo* have several anticancer effects on breast cancer, including growth arrest, the induction of apoptosis, and cellular differentiation [20],[25]–[27].

In addition to their efficacy as preclinical monotherapy in breast cancer cells, HDACis enhance sensitivity to radiotherapy [20] and cytotoxic agents [28]. For example, the combination of vorinostat and TRAIL resulted in significant growth inhibition when compared with either treatment alone in mice bearing TRAIL-resistant tumor xenografts [28]. Various HDACis, including valproic acid, trichostatin A, and entinostat, have been shown to play a role in overcoming resistance to therapies. In this respect, HDACis can be exploited for overcoming resistance to HER2-targeted therapies [29]. Also, HDACis are well accepted for their anticancer activities through promoting re-expression of silenced genes such as *ESR1 in vitro* and *in vivo* [30],[31]. Moreover, re-expression of *ESR1* re-sensitized breast cancer cells to the ER-targeted therapy tamoxifen *in vitro* [24],[32]. Paradoxically, HDACis have non-selective effects on non-histone proteins, which might cause opposite effects. For example, in ER-positive breast cancer cells, ER-α expression decreased upon treatment with vorinostat. This effect can be due to increased acetylation levels of heat shock proteins, which are known to stabilize the ER-α protein and inhibit its degradation [33]. Despite these opposite effects, however, the combination of HDACis and endocrine therapy acted synergistically in ER-positive models [34].

## FDA approved epi-drugs in oncology

Azacitidine and decitabine are both approved by the United States Food and Drug Administration (FDA) for the treatment of myelodysplastic syndrome. Azacitidine is administered by subcutaneous or intravenous injections once daily for 7 days followed by 21 days without treatment. Decitabine is given intravenously thrice daily for 3 consecutive days followed by 4 days without treatment. In the setting of myelodysplastic syndrome, both treatments provide an objective response (complete + partial response) of 16 to 17% compared with no response in untreated controls. Both regimens show comparable toxicity profiles, with myelosuppression, gastrointestinal complaints and constitutional symptoms the most common side effects [35].

Vorinostat and romidepsin are FDA-approved HDACis for the treatment of cutaneous T-cell lymphoma; in addition, romidepsin is approved for the treatment of peripheral T-cell lymphoma [36]. Vorinostat 400 mg orally once daily induced objective responses in approximately 30% of patients [37]. The most common adverse events include myelosuppression, gastrointestinal side effects and fatigue [37]. Administration of romidepsin as a 4-hour infusion on days 1, 8, and 15 of a 28-day cycle with a starting dose of 14 mg/m^2^ resulted in an objective response in 34% of patients with cutaneous T-cell lymphoma [38],[39] and in 38% of patients with peripheral T-cell lymphoma [40]. Side-effects are comparable to those of vorinostat.

## Efficacy of epi-drugs in breast cancer patients

The efficacy of DNMTis and HDACis in breast cancer was evaluated in 21 phase I and II studies that enrolled 303 patients with breast cancer (Table 1). In 11 of these studies (n = 87 patients) epi-drugs were administered to the patient either as monotherapy or in combination with another epi-drug. Most of these studies were phase I studies (64%) in advanced solid tumors and were, therefore, not primarily aimed to evaluate anti-tumor efficacy, including only few patients who were, in general, heavily pre-treated. Nevertheless, epi-drugs in breast cancer have consistently shown very limited anti-tumor efficacy on their own. Of 87 patients receiving epi-drugs as monotherapy, objective responses were observed in only 9 (10%). The limited efficacy of epi-drugs at the maximum tolerated dose suggests that they are not well suited as monotherapy in breast cancer. However, biological efficacy at the epigenetic level was observed; for instance, pre- and post-treatment tumor biopsies showed significant reduction in tumor DNA methylation after decitabine monotherapy [41].

**Table 1:** **Efficacy of epi-drug monotherapy and combination therapies in breast cancer patients**

Epi-drug	Phase	Co-treatment	Number of patients	OR/CBR	Reference
**Monotherapy**					
Azacitidine	I	None	11	7/NA	[42]
Azacitidine plus valproic acid	I		4	0/0	[43]
Decitabine	I		4	0/NA	[41]
Fazarabine	I		3	1/1^a^	[44]
	II		14	0/0	[45]
Phenylbutyrate	I		5	0/NA	[46]
Vorinostat	II		14	0/3	[47]
	II		3	0/0	[48]
	II		26	1/1	[49]
	Biomarker study		-	NA	[50]
Vorinostat plus Decitabine	I		3	0/0	[51]
**Total**			**87**	**9 (10%)/NA**	
**Combination therapies**					
Azacitidine	I	Erlotinib	1	0/1	[52]
Decitabine	I	Carboplatin	5	0/NA	[53]
Entinostat	II	Exemestane	64^b^	4/18	[54]
Valproic acid	II	5-Fluoruracil, epirubicin and cyclophosphamide	15	9/NA	[55]
	I	Followed by epirubicine	10	3/7	[56]
Valproic acid plus hydralazine	II	Standard chemotherapy	3	0/0	[57]
	I	Doxorubicin plus cyclophosphamide	16	13/NA	[58]
Vorinostat	I	Doxorubicin	5	1/1	[59]
	I-II	Paclitaxel plus bevacizumab	54	26/42	[60]
	II	Tamoxifen	43	8/17	[35]
**Total**			**216**	**64 (30%)/NA**	

^a^Clinical response. ^b^An additional 67 patients were randomized to exemestane plus placebo. CBR, objective response + stable disease >6 months); OR, objective response (partial + complete remission); NA, not available.

Given that epi-drugs can alter the expression of therapeutic targets, this led to the hypothesis that they should especially be administered as a (re-)sensitizer for drugs to which intrinsic or acquired resistance exists. This novel approach has rendered promising results in other tumor types in clinical trials. Decitabine was shown to allow the re-expression of the copper transporter CTR1, which plays a role in cellular platinum-uptake, in patients with solid tumors and lymphoma [41], and restore sensitivity to platinum-based chemotherapy in ovarian cancer [61],[62]. A combination of epi-drugs with cytotoxic or targeted therapies, such as ER-targeted therapy, was evaluated in 10 phase I/II studies in 216 breast cancer patients. The largest study so far is a phase II study in which 130 metastatic breast cancer patients were randomized to exemestane plus placebo (n = 66) or exemestane plus entinostat (n = 64) [54]. These patients had earlier progressed on a nonsteroidal aromatase inhibitor. The combination of exemestane plus entinostat significantly improved progression-free survival (4.3 versus 2.3 months) and overall survival (28.1 versus 19.8 months) [54]. In another phase II study in 43 patients with metastatic breast cancer who progressed on at least one prior line of endocrine therapy, vorinostat 200 mg twice daily was combined with tamoxifen [35]. In this study, the objective response rate was 19% and the clinical benefit rate (objective response or stable disease >6 months) was 40%*.* Baseline high HDAC2 levels correlated with response, which may prove valuable as a predictive biomarker to select patients for treatment with HDACis. Finally, in a phase I/II study in 54 patients with metastatic breast cancer, vorinostat 200 to 300 mg twice daily on days 1 to 3, 8 to 10, and 15 to 17 was added to paclitaxel plus bevacizumab [60]. This combination resulted in a 49% objective response rate (partial + complete remission) and 78% clinical benefit rate (objective response + stable disease >6 months). Serial biopsies, available from seven patients, showed an increase in acetylation of heat shock protein 90 and α-tubulin.

Although there is preclinical evidence for enhanced efficacy of HER2-targeted therapies when combined with epi-drugs, results from clinical studies are awaited.

In conclusion, epi-drugs have limited anti-tumor efficacy in breast cancer patients at the maximum tolerated dose when administered as monotherapy, but can be administered safely. However, expected epigenetic changes, such as decreased tumor DNA methylation [41], increased histone acetylation [60], and upregulation of gene expression [58], are observed after their administration in clinical breast cancer studies. Current studies suggest a potential role for epi-drugs in combination with chemotherapeutics and targeted therapies to enhance or restore the sensitivity to these drugs.

## Current breast cancer trials evaluating epi-drugs

Ongoing trials increasingly apply epi-drugs to specific subgroups rather than to the general breast cancer population. Much work is performed on (re-)sensitization of endocrine-resistant tumors to endocrine therapy. In patients with triple-negative or hormone-refractory metastatic breast cancer, azacitidine is combined with entinostat; although the response rate is the primary endpoint in this study, the effects on ER and PR expression will be evaluated as secondary endpoints (NCT01349959). A novel, non-invasive way to measure ER expression is by molecular imaging using positron emission tomography (PET) and ^18^ F-fluoroestradiol (FES) as a tracer [63]. This tool facilitates the assessment of ER expression during treatment. In a study, hormone-refractory patients are being treated with daily vorinostat for 2 weeks, followed by a treatment with an aromatase inhibitor for 6 weeks (NCT01153672). Cycles are repeated every 8 weeks until progression. As a secondary endpoint, changes in ER expression will be measured using serial FES-PET imaging. Panobinostat and decitabine are also being evaluated to sensitize triple-negative breast cancer patients to endocrine therapy in phase I/II studies (NCT01194908, NCT01105312).

The use of DNMTis and HDACis as chemo-sensitizers is also being evaluated in various breast cancer trials (for example, NCT00748553, NCT00368875). Among the evaluated combinations are azacitidine with Nab-paclitaxel (Abraxane®, Abraxis Bioscience, Los Angeles, CA, USA), valproic acid with FEC, and vorinostat with paclitaxel plus bevacizumab. Finally, sensitization to HER2-targeted therapy will be evaluated in a limited number of studies. One phase I/II study evaluated 200 mg vorinostat twice daily on days 1 to 14 combined with trastuzumab 6 mg/kg once every 3 weeks. This study enrolled 16 patients and was terminated due to low response rate (NCT00258349). Another study will evaluate the safety and efficacy of vorinostat combined with the tyrosine kinase inhibitor lapatinib (NCT01118975). Also, several studies using panobinostat to sensitize breast cancer to trastuzumab (NCT00788931, NCT00567879), and lapatinib (NCT00632489) have recently been completed and results are awaited. All trials were phase I or II. An overview of ongoing trials with DNMTis and/or HDACis in breast cancer is provided in Table 2.

**Table 2:** **Overview of current clinical trials evaluating DNMT-inhibitors and HDAC-inhibitors in breast cancer**

Drug	Condition	Co-treatment	Primary outcome measure	***N***	Phase	Status	NCT number
**DNMT inhibitor**							
Azacitidine	Advanced BC	Entinostat^a^	Objective response rate	60	II	R	01349959
	Advanced/metastatic BC	Nab-paclitaxel		45	I/II	R	00748553
Decitabine	Advanced/metastatic TNBC	Panobinostat^b^ (±tamoxifen)	The maximum tolerated dose of decitabine and panobinostat	60	I/II	R	01194908
FdCyd	Solid tumors, including BC	Tetrahydrouridine	To determine the safety of FdCyd	20	I	R	01479348
FdCyd	Solid tumors, including BC	Tetrahydrouridine	To determine PFS and/or response rate of FdCyd plus tetrahydrouridine	185	I	R	00978250
EGCG	Newly diagnosed BC	-	To determine whether EGCG can affect proliferation rate and induce apoptosis	20	II	R	00949923
	Newly diagnosed BC	-	To evaluate the effects of EGCG on various biomarkers	32	II	A	00676793
	Stage I-III BC	-	To determine the safety and maximum tolerated dose of EGCG	40	I	A	00516243
HDAC inhibitor							
Vorinostat	BC	Lapatinib	Clinical benefit rate	47	I/II	R	01118975
	Recurrent/metastatic BC	-	To evaluate the safety of vorinostat	49	I/II	A	00416130
	Advanced BC	Capecitabine	The maximum tolerated dose, safety, and efficacy of vorinostat plus capecitabine	47	II	U	00719875
	Local recurrent/metastatic BC	Paclitaxel/bevacizumab	The maximum tolerated dose, and objective response rate of vorinostat in combination with paclitaxel/bevacizumab	58	I/II	U	00368875
	Metastatic BC	Ixabepilone	Dose limiting toxicity	56	I	A	01084057
	Hormone-refractory BC	Aromatase inhibitor	Clinical benefit rate	14	II	R	01720602
	Locally advanced BC	Paclitaxel/trastuzumab	To determine the recommended phase II dose	54	I/II	U	00574587
	Hormone-refractory BC	Aromatase inhibitor	Clinical benefit rate	20	II	R	01153672
	Newly diagnosed BC	Nab-paclitaxel/carboplatin	Pathologic complete response rate	74	II	A	00616967
	HIV + with solid tumor, including BC	Paclitaxel/carboplatin	Maximum tolerated dose	66	I	R	01249443
	Brain metastases, including from BC	Paclitaxel/carboplatin plus radiotherapy	Maximum tolerated dose	24	I	A	00838929
Entinostat	Locally recurrent/metastatic ER + BC, or NSCLC	±Exemestane	Pharmacokinetics of entinostat in fasted and fed subjects	28	I	R	01594398
	Newly diagnosed TNBC	Anastrozole	Safety, tolerability and recommended phase II dose (phase I cohort); change in proliferation, ER/PR expression (phase II cohort)	41	I/II	R	01234532
	HER2-positive metastatic BC	Lapatinib	Recommended phase II dose (phase I cohort); objective response rate (phase II cohort)	70	I/II	R	01434303
	Advanced BC	Azacitidine^a^	Objective response rate	60	II	R	01349959
Panobinostat	Metastatic TNBC	Letrozole	Maximum tolerated dose, adverse events (phase I cohort); response rate (phase II cohort)	48	I/II	R	01105312
	Advanced/metastatic TNBC	Decitabine^b^ (±tamoxifen)	The maximum tolerated dose of decitabine and panobinostat	60	I/II	R	01194908
	HER2-negative locally recurrent/metastatic BC	-	Objective response rate	118	II	A	00777049
VPA	Newly diagnosed locally advanced/metastatic BC	FEC	Pathologic response rate	55	II	R	01010854
	Newly diagnosed BC	-	To determine whether VPA levels correlate with leukocyte and tumor histone acetylation	33	NA	R	01007695
Depsipeptide	Solid or hematologic malignancy, including BC	-	Safety, tolerability, maximum tolerated dose and pharmacokinetics	132	I	R	01638533

*N* = estimated enrolment. Status: A = active, not recruiting; C = completed; R = recruiting; U = unknown. ^a,b^Cross-referenced within table. BC, breast cancer; DNMT, DNA methyltransferase; EGCG, epigallocatechin-3-gallate; ER, estrogen receptor; HDAC, histone deacetylase; NA, not applicable; NSCLC, non small-cell lung cancer; PFS, progression-free survival; PR, progesterone receptor; TNBC, triple-negative breast cancer; VPA, valproic acid.

## Epigenetic editing

Despite the above-described promises, epi-drugs affect genes in a genome-wide manner, as well as inhibit writers and erasers, which generally also modify non-chromatin proteins. Such aspecific mechanisms of action result in unwanted effects, including upregulation of prometastatic genes [64] or of genes encoding drug resistance-associated proteins [65]. To fully exploit the reversible nature of epigenetic mutations while avoiding unwanted effects, epigenetic therapy can be improved using gene targeting approaches: by fusing a writer or eraser of a particular epigenetic mark to a self-engineered DNA binding domain, rewriting of the epigenetic signature of a selected target gene (epigenetic editing) is achieved [6]. To obtain sequence-targeted DNA binding, zinc finger proteins (ZFPs), triplex forming oligos, transcription activator-like effectors (TALEs), or catalytically inactive Cas proteins of the clustered regularly interspaced short palindromic repeats system [66],[67] can be fused to the catalytic domains of epigenetic enzymes (epigenetic effector domains) [6],[68] or to epi-drugs [69]. The epigenetic effector domain of an epigenetic editing tool will subsequently overwrite epigenetic modifications at the targeted gene. Because of cellular epigenetic maintenance processes, edited epigenetic modifications (or sets thereof) might remain on the DNA or histone tails, even after removal of the epigenetic editing tool. Moreover, written epigenetic modifications can spread along the target gene [70],[71] due to subsequent recruitment of endogenous epigenetic enzymes [72],[73]. Interestingly, adequately rewritten epigenetic modifications might be inherited by subsequent cell generations [74], thereby allowing permanent changes to genome functioning without changing genomic sequences. Altogether, epigenetic editing provides a promising novel avenue to interfere with gene expression levels in a persistent manner.

As epigenetic editing targets a gene directly at the DNA level, this targeting of generally two copies of DNA offers advantages over targeting multiple copies of or different isoforms of proteins or RNA. Moreover, since RNA and protein molecules are constantly being expressed, their sustained inhibition requires continuous administration of inhibitors or potentially harmful integration of the (RNA interference) transgene expression cassette into the host genome. Epigenetic editing allows a hit-and-run approach to directly silence the source of the RNA production. Alternatively, for upregulation of a gene’s expression level, epigenetic editing tools can be engineered to remove epigenetic repressive marks and/or induce activating marks at selected loci. Such overwriting of repressive signatures will allow transcription of alternative isoforms to take place, in their natural ratios. For example, for upregulation of tumor suppressor genes that are frequently silenced by epigenetic mutations, activating the expression from their endogenous DNA loci better mimics nature than administration of ectopic cDNA expression constructs, which result in overexpression of only one isoform of a gene.

Proofs of concept for locus-specific epigenetic overwriting have been described for numerous epigenetic effector domains [6]. To date, 10 papers describe epigenetic editing on endogenous genes. Using engineered ZFPs, we showed that targeted DNA methylation is instructive in gene expression downregulation (for example, of *MASPIN* [75], *VEGF-A* [76], and EpCAM [77]). Interestingly, targeted DNA demethylation could be induced, which was effective in upregulating the expression of the targeted gene *ICAM-1* [78]. We also demonstrated that writing the repressive histone methylation modification H3K9me2 on the *Her2/neu* gene induced Her2/neu protein downregulation, which in turn inhibited cancer cell growth [79]. Our findings for an overexpressed oncogene validated results of an earlier report on downregulation of VEGF-A [70]. Moreover, targeted DNA methylation of the *SOX2* promoter prevented growth of breast cancer cells, also upon removal of the epigenetic writer [75]. Others recently joined the field and demonstrated the power of epigenetic editing as a unique research tool in addressing epigenetic control of gene expression regulation [80],[81]. Interestingly, active DNA demethylation has also been demonstrated using engineered TALE-TET2 fusions [82] or by fusing a DNA repair enzyme to engineered ZFPs [83]. As targeting of genes has recently become widely feasible [84], epigenetic editing opens new avenues towards 'the druggable genome', and since multiplex gene targeting is currently feasible, cancer therapy approaches might also benefit from such progress.

## Conclusion

Epigenetic mutations, including aberrant DNA methylation and histone modifications, are associated with breast cancer development and therapy resistance. Aberrant DNA methylation and histone acetylation can be reversed by DNMTis and HDACis. Several DNMTis and HDACis are FDA approved, albeit not so far for the treatment of patients with breast cancer. These drugs can induce apoptosis, alter gene expression, and reverse therapy resistance in preclinical models. In clinical studies, DNMTis and HDACis have shown very modest anti-tumor activity as monotherapy, although effects on gene expression can be observed. Current clinical trials, therefore, mainly focus on the combination of these drugs with chemotherapeutics and targeted therapies. Despite their promise, a disadvantage of DNMTis and HDACis is their genome-wide function and non-chromatin effects. Epigenetic editing of a single gene results in gene expression modulation, and thereby fully exploits the reversibility of epigenetic modifications as therapeutic targets while reducing off-target effects. Epigenetic editing and other targeted approaches thus provide alternatives to current epigenetic therapies for breast cancer.

## Authors’ original submitted files for images

Below are the links to the authors’ original submitted files for images. Authors’ original file for figure 1

## Abbreviation

DNMT: DNA methyltransferase
DNMTi: DNA methyltransferase inhibitor
ER: Estrogen receptor
FDA: Food and Drug Administration
FES: ^18^ F-fluoroestradiol
H: Histone
HAT: Histone acetyltransferase
HDAC: Histone deacetylase
HDACi: Histone deacetylase inhibitor
PET: Positron emission tomography
PR: Progesterone receptor
TALE: Transcription activator-like effector
TRAIL: Tumor necrosis factor related apoptosis-inducing ligand
ZFP: Zinc finger protein
