# Supplementary figures and images for: Current and upcoming approaches to exploit the reversibility of epigenetic mutations in breast cancer
Source: Breast Cancer Res. 2014 Jul 29;16:412. doi: 10.1186/s13058-014-0412-z (PMC4303227; doi:10.1186/s13058-014-0412-z)

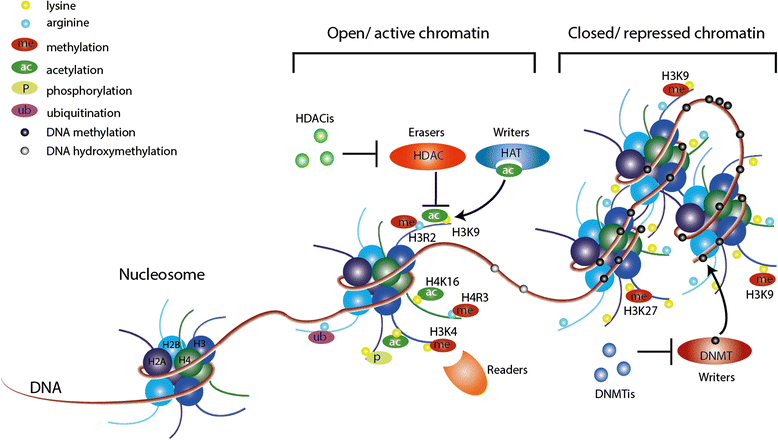

Supplement: Supplementary file 1 — Authors’ original file for figure 1 [file 13058_2014_412_MOESM1_ESM.gif]
